# A comprehensive review of the ethnomedicine, phytochemistry, pharmacological activities of the genus *Kniphofia*

**DOI:** 10.1080/13880209.2022.2085753

**Published:** 2022-06-14

**Authors:** Gashaw Nigussie, Metasebia Tegegn, Dessalegn Abeje, Haregua Melak

**Affiliations:** a Department of Biotechnology and Bioinformatics, Armauer Hansen Research Institute, Addis Ababa, Ethiopia; b Department of Immunology, Armauer Hansen Research Institute, Addis Ababa, Ethiopia

**Keywords:** Ethnomedicinal uses, bioactive compounds, anthraquinones, antimalarial activity, anti-HIV-1 activity

## Abstract

**Context:**

*Kniphofia* (Asphodelaceae) is found mainly in South Africa and Tropical Africa. Malaria, hepatitis B, blood purifier, cancer, eczema, and female infertility have all been traditionally treated using this genus.

**Objective:**

The current review provides a complete and up-to-date compilation of documented traditional medicinal uses, phytochemicals, and pharmacological activities of the genus.

**Method:**

Relevant literature was collected by searching the major electronic scientific databases including PubMed, Science Direct, Web of Science, and Google Scholar using appropriate keywords ethnomedicinal studies, phytochemical investigations, and pharmacological activities of *Kniphofia* species. The search strategy included all articles with descriptors that were available until November 30, 2021. Only published works in English were used for this study. The data were collected using textual descriptions of the studies, tabulation, grouping, and figures.

**Result:**

At present, more than 40 compounds have been isolated from different parts of *Kniphofia* species. The major compounds isolated from the *Kniphofia* species are monomeric anthraquinones and dimeric anthraquinones. Pharmacologically the extracts and isolated compounds showed antioxidant, antimalarial, antiproliferative, anti-HIV-1, anti-leukotriene, and cytotoxic activity. The genus afforded exemplary drug leads such as knipholone and knipholone anthrone with anti-HIV-1, antimalarial and cytotoxicity activity.

**Conclusions:**

*Kniphofia* species have traditionally been used to treat a variety of diseases. Pharmacological actions of phytochemicals were shown to be promising. Despite this, considering the genus's inclusion on the red data list of South Africa, it deserves more attention. In order to find novel drug candidates, more studies on promising crude extracts and compounds are needed.

## Introduction

Plants, according to the locals, have nutritional, therapeutic, and mystical properties. Medicinal plants play an important role in local communities' healthcare systems as major components of medicine, particularly among the rural population (Nigussie 2021). Plant knowledge and application are strongly linked to ethnic cultures. The distribution, taxonomic variety, and abundance of medicinal plants vary based on location and climatic circumstances, and ethnomedicinal healing systems vary between societies (Farooq et al. 2019). The World Health Organisation (WHO) report shows that over nearly 80% of the world's population uses herbal plants to cure human ailments (WHO 2013). The report also demonstrates that medicinal plants are being studied as an alternative therapy and support for health-care activities. Traditional medicine incorporates medical parts of indigenous knowledge that have been passed down through generations prior to the development of modern medicine.

Traditional medicine is defined by the WHO as the sum of all skills, knowledge, and practices based on theories, beliefs, and indigenous experiences of various cultures and used in health care for the prevention, diagnosis, improvement, and treatment of mental and physical disease (WHO 2013). Traditional medicine which is mainly based on plants has been frequently confirmed by phytochemical investigations, pharmacological studies and clinical tests initiating further studies on medicinal plants in different parts of the world (Nigussie et al. 2021). Traditional medicines, on the other hand, can have adverse side effects, thus additional studies are needed to ensure the efficacy and safety of traditional medicine and methods employed by traditional medicine practitioners and consumers. WHO has launched a nine-year strategic plan to support member states in developing proactive strategies and implementing action plans that strengthen the role of traditional medicine in keeping populations healthy (WHO 2013).

*Kniphofia* is a genus of plants named after Johann Hieronymus Kniphof, a German botanist (1704-63) (Armitage 2011). The genus *Kniphofia* Moench, commonly known as ‘red hot pokers’, belongs to the family Asphodelaceae in the sub-family Asphodeloideae (Kubitzki et al. 1998). According to Codd (1968), the genus *Kniphofia* contains 70 species, 45 of which are found in South Africa, 1 in the Arab Republic of Yemen, 2 in Madagascar, and 23 in Tropical Africa, including 7 in Ethiopia. However, Ramdhani et al. (2006) later acknowledged that the genus contains approximately 71 species by incorporating *Kniphofia monticola*, which was not previously included by Codd (1968). Several *Kniphofia* species have been utilised extensively and regularly by society and traditional healers for a variety of diseases. The leaves, stems, and roots are used in the formulation of traditional medicine. Various biological responses of *Kniphofia* indicate an extensive range of plant-derived compounds in various classes of chemical groups.

The World Health Organisation's traditional medicine policy (WHO 2013) stated “traditional medicine and complementary products, practices, and practitioners will continue to be in high demand. In the meantime, there is a knowledge documentation gap. According to the current publication status, no comprehensive review study on the many features linked with the *Kniphofia* genus has been published. Therefore, the botanical description, reproduction, economic and ethnomedicinal usage, conservation, phytochemistry, and pharmacological applications of the *Kniphofia* genus are all covered in this review study.

## Review methodology

This review adheres to the three-step review approach (Toyang and Verpoorte 2013). These include searching the literature, selecting relevant articles, and checking species names. The most widely used search tools or databases such as Google Scholar, PubMed, Scopus, Science Direct and Web of knowledge for the search terms: *Kniphofia* species, ethnomedicinal studies, phytochemical investigations, and pharmacological activities. The search strategy included all articles with descriptors that were available until November 30, 2021. Only published works in English have been used on this study. The data were collected using textual descriptions of the studies, tabulation, grouping, and figures. The worldwide plant name index (https://www.ipni.org) and the Kew Botanical Garden plant name database (https://www.kew.org) were used to check species names.

### Botany and distribution of the genus *Kniphofia*

*Kniphofia* is a perennial, acaulescent, and herbaceous genus with a single or branched thick rhizome and a thick well-developed stem that can be caespitose or solitary. The leaves are arranged in a basal rosette, generally in 4 or 5 ranks, but occasionally in 2, are linear, and taper gradually to the tip, and are frequently keeled. The leaf margin varies in texture from smooth to finely serrulate. Inflorescence peduncles are terminal, stout, erect, sub-equal to the leaves, simple or rarely branching, necked save for infrequent sterile bracts below the inflorescences, and inflorescences are sub-capitate racemes of usually numerous flowers, dense or lax. The bracts are scarlet or brown in colour, persistent, and longer than the pedicels. The pedicels are short to almost absent and articulated at the apex and flowers are spreading or pendulous with white, yellow or various shades of red. The perianth is tubular, campanulate to cylindrical or somewhat funnel-shaped and short, sub-equally lobed. The stamens are usually as long as or longer than the perianth at anthesis and the ovary is sessile, ovoid, 3-locular with many ovules in each locule. The fruits are globose to ovoid often 3-angled with loculicidal dehiscence and seeds are somewhat flattened, acutely 3-angled or winged (Hedberg et al. 1997). The underground component of *Kniphofia* is made up of a strong rhizome and fibrous, meaty roots. The rhizome divides in certain species, generating groups of stems, while others have stems that are more or less solitary.

**Figure 1.**
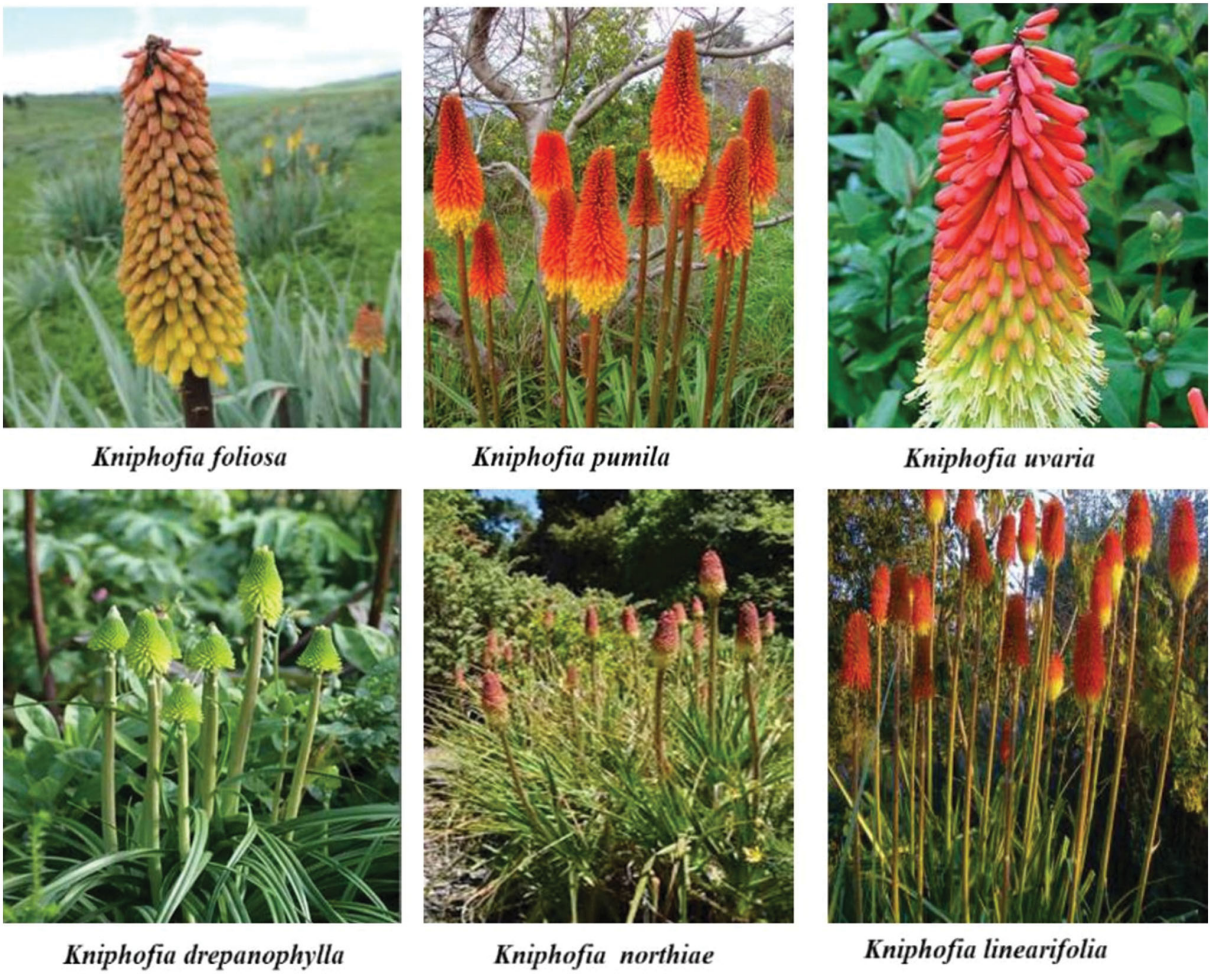
Images of some representative samples of *Kniphofia* species.

According to Ramdhani et al. (2006), the genus *Kniphofia* has six centres of diversity, five of which are endemism centres. The South African Centre is the most important in terms of species diversity and endemism, and it is also the largest. According to Marais (1973), the genus *Kniphofia* varies in size depending on the location and availability of water, and it can be found in a variety of environments ranging from low and wet savannah grassland to montane and alpine vegetation. In tropical and East Africa, *Kniphofia* has a strong afromontane grassland affinity. In South Africa, it is found from high altitudes to coastal habitats, with the most species regions being afromontane grasslands. It is, thus, not considered to be an afromontane element, but rather an afromontane associate. Five major evolutionary lineages were identified using cpDNA sequence data (trnT-L spacer), four of which are southern African. The fifth lineage was represented by material from Madagascar, tropical and East Africa (Ramdhani et al. 2006). In Ethiopian flora, seven *Kniphofia* species, *Kniphofia foliosa* Hochst., *Kniphofia hildebrandtii* Cufod., *Kniphofia insigns* Rendle., *Kniphofia isoetifolia* Hochst., *Kniphofia pumila* (Ait) Kunth., *Kniphofia schimperi* Baker., and *Kniphofia thomsonii* Baker were identified (Hedberg et al. 1997). Of those *Kniphofia foliosa*, *Kniphofia hildebrandtii*, *Kniphofia insignis*, *Kniphofia isoetifolia*, and *Kniphofia schimperi* are all endemic to Ethiopia, whereas *Kniphofia pumila* and *Kniphofia thomsoni* are widely distributed from West Africa to Eastern and Central Africa. *Kniphofia thomsoni* is found in Kenya, Uganda, and Tanzania, particularly on Mount Kilimanjaro (Marais 1973). Some of the representative samples of *Kniphofia* species are presented in Figure 1 (Codd 1968; Whitehouse 2002; Brown et al. 2009).

### Reproduction

*Kniphofia* can be sexually propagated by seed. However, in all species, the low number of sexually reproducing plants may have an impact on the number of seedlings produced from seeds, which may have a negative impact on the plants' long-term survival. Furthermore, because all *Kniphofia* species are obligatory outcrossers, a decrease in new seedlings could lead to a decrease in seed production due to gametophytic self-incompatibility. It can also reproduce asexually by dividing underground stems known as short rhizomes into ramets, which have the ability to be physiologically independent. As a result, even though it generates all genets in an area with the same genetic makeup due to proliferation and later fragmentation into clones, the asexual form of reproduction known as vegetative reproduction contributes more to population expansion (Teklehaymanot 2001).

### Economic and ethnomedicinal importance

The genus is well-known for its ornamental value due to its colourful flowers, and it is used in horticulture and is planted in both home and botanical gardens. *Kniphofia* species found in nature are major pollen and nectar sources for honeybees (Fichtl and Adi 1994). An infusion of the roots is used to relieve or treat chest disorder and *Kniphofia parviflora* is reported to have been made into a traditional snake repellant (Bringmann et al. 2008). In Ethiopian traditional medicine, the roots of *Kniphofia foliosa* are used to relieve abdominal cramps (Wube et al. 2005). The use of the genus *Kniphofia* in traditional medicine is limited to few species which is summarised in Table 1.

**Table 1.** Botanical distribution and traditional medicinal uses of the genus *Kniphofia*.

Species	Disease treated	Plant organs used	Preparation and application	Distribution	Ref
*Kniphofia foliosa*	Cervical and breast	Roots	Dry roots are pounded and the powder is mixed with honey.	Ethiopia	Tesfaye et al. 2020
*Kniphofia isoetifoli*	Gonorrhoea	Roots	Concoction, crushing and powdering and taken orally	Ethiopia	Bizuayehu and Garedew 2018
	Hepatitis B	Roots	Fresh or dried roots concocted, crushed, decocted	Ethiopia	Yineger et al. 2013
*Kniphofia caulescens*	Headache, painful eyes	Root bulb	Crush & add water	South Africa	Mugomeri et al. 2016
	Blood purifier		Not Reported	South Africa	Van Vuuren & Frank 2020
*Kniphofia northiae*	Period pains; menorrhagia	Stems	Decoction taken orally	South Africa	Moteetee and Kose 2016
*Kniphofia drepanophylla*	Ringworm, wounds, pimples, acne and eczema.	Rhizomes (root)	Dry, grind and mix with red oak or use alone in water. Apply on affected parts.	South Africa	Josia 2013
*Kniphofia sumarae*	Malaria	Roots	Not Reported	Yemen	Al-Musayeib et al. 2012
*Kniphofia pumila*	Evil eye	Bulbs	Soak it in water with leaves of Rumex nervosus and wash body with it	Ethiopia	Teklay et al. 2013
*Kniphofia reflexa*	High relapsing fever	Rhizomes	Not reported	Cameroon	Sema et al. 2018
*Kniphofia uvaria*	Dysmenorrhoea	Rhizome	Not reported	South Africa	Steenkamp 2003
*Kniphofia linearifolia*	To treat infertility in women	Roots	The powdered root is consumed by mixing it with food.	Zimbabwe	Bosch 2008

### Conservation

Many *Kniphofia* species are in urgent need of conservation because a high number of South African species are included in the red data list of Hilton-Taylor (1996). Scott-Shaw (1999), identified 17 *Kniphofia* taxa in Kwazulu Natal (South Africa) and surrounding areas that are considered endangered. The endemic *Kniphofia hildebrandtii* in Ethiopia likewise requires special attention due to its biologically limited distribution and location in very venerable grassland that is exploited for livestock grazing. Additionally, *Kniphofia insignis*, which is found in wetland habitats, requires special attention because the community is converting wetland ecosystems to agriculture, so that it will not have refuge places to escape (Teklehaymanot 2001).

### Phytochemistry

**Figure 2.**
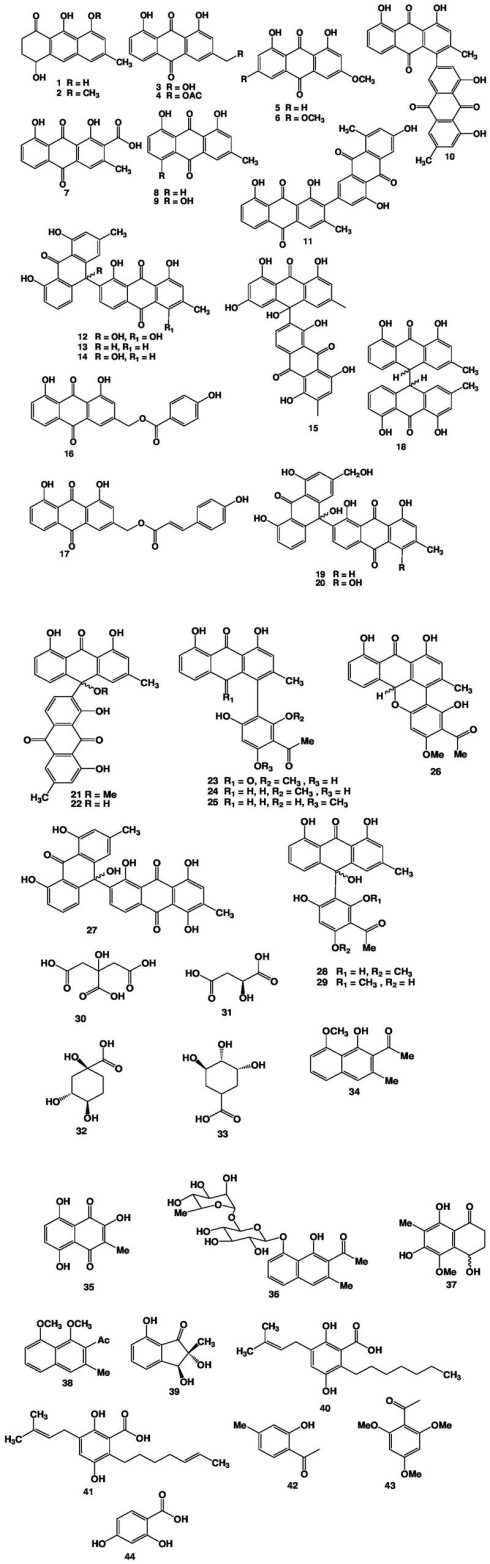
Structure of isolated compounds from *Kniphofia* species.

The genus *Kniphofia* is comprehensively studied for its chemical constituents and till now, more than 40 compounds from different chemical classes have been identified. These phytochemicals mainly contain anthraquinones, naphthalene derivatives, organic acids, indane derivatives and miscellaneous group of compounds. Monomeric anthraquinone, dimeric anthraquinone and Phenyl anthraquinones and anthrones are the major constituents isolated from the majority of the *Kniphofia* species. Many of the isolated compounds were also evaluated for their bioefficiency. The methods used for isolating new compounds for the plants of *Kniphofia* species include serial extraction, bioassay guided extraction, high performance liquid chromatography (HPLC), apart from the successive fractionation using different polarity solvents and column chromatography. Activity guided bioactive compound isolation is currently gaining attention because of the increased demand for the use of traditional medicine as an alternative and complementary medicine (WHO 2013). The root of the plants in the genus was frequently considered for investigation. Indeed, *Kniphofia* species are mostly found in Tropical Africa and South Africa, which explains why there aren't many compounds isolated from this genus. This could be due to a number of factors, including plant availability, material shortages, a lack of skilled manpower, and the tedious nature of the work. The summaries for the phytochemical investigation are presented in Table 2 and Figure 2 depicts the structures of those compounds.

**Table 2.** Recently isolated compounds from *Kniphofia* species.

Compound	Species	Collection area	Plant organ investigated	Extraction method used	Ref
**Pre anthraquinones**					
Aloesaponol III (**1**)	*Kniphofia foliosa*	Ethiopia	Stem	SE, TLC and CC	Yenesew et al. 1994
Aloesaponol III-8-methyl ether (**2**)	*Kniphofia foliosa*	Ethiopia	Stem	SE, TLC and CC	Yenesew et al. 1994
**Monomeric anthraquinones**					
Aloe-emodin (**3**)	*Kniphofia foliosa*	Ethiopia	Leaves, flowers, fruits	SE, TLC and CC	Berhanu et al. 1986
	*Kniphofia insignis*	Ethiopia	Flowers		
	*Kniphofia isoetifolia*	Ethiopia	Flowers		
	*Kniphofia schimperi*	Ethiopia	Flowers		
	*Kniphofia thomsonii*	Kenya	Roots	SE, TLC and CC	Achieng 2009
	*Kniphofia ensifolia*	South Africa	Whole parts	BGE, SE, TLC and HPLC	Dai et al. 2014
Aloe-emodin acetate (**4**)	*Kniphofia foliosa*	Ethiopia	Leaves, Flowers, Fruits	SE, TLC and CC	Berhanu and Dagne 1984; Berhanu et al. 1986
	*Kniphofia isoetifolia*	Ethiopia	Flowers	SE, TLC and CC	Berhanu et al. 1986
	*Kniphofia thomsonii*	Kenya	Roots	SE, TLC and CC	Achieng 2009
Chrysophanol (**5**)	*Kniphofia foliosa*	Ethiopia	Rhizomes, leaves,	SE, TLC and CC	Berhanu et al. 1986
	*Kniphofia insignis*	Ethiopia	Rhizomes	SE, TLC and CC	Berhanu et al. 1986
	*Kniphofia isoetifolia*	Ethiopia	Rhizomes, leaves, flowers, Roots	SE, TLC and CC	Berhanu et al. 1986; Meshesha et al. 2017
	*Kniphofia pwnila*	Ethiopia	Rhizomes	SE, TLC and CC	Berhanu et al. 1986
Chrysophanol (**5**)	*Kniphofia schimperi*	Ethiopia	Rhizomes, flowers	SE, TLC and CC	Berhanu et al. 1986
	*Kniphofia thomsonii*	Kenya	Roots	SE, TLC and CC	Achieng 2009
Chrysophanol (**5**)	*Kniphofia reflexa*	South Africa	Rhizomes	FC, CC, HPLC and PTLC	Dai et al. 2014
	*Kniphofia ensifolia*	South Africa	Whole parts	BGE, SE, TLC and HPLC	Dai et al. 2014
Physcion (**6**)	*Kniphofia thomsonii*	Kenya	Roots	SE, TLC and CC	Achieng 2009
Chrysophanic acid (**7**)	*Kniphofia caulescens*		Roots	SE, TLC and CC	Yenesew et al. 1988
	*Kniphofia foliosa*	Ethiopia	Leaves, rhizomes, roots and fruits	SE, TLC and CC	Berhanu and Dagne 1984; Berhanu et al. 1986; Yenesew et al. 1988
	*Kniphofia insignis*	Ethiopia	Rhizomes	SE, TLC and CC	Berhanu et al. 1986
Chrysophanic acid (**7**)	*Kniphofia isoetifolia*	Ethiopia	Flowers, leaves,	SE, TLC and CC	Berhanu et al. 1986
	*Kniphofia linearifolia*	Ethiopia	Roots	SE, TLC and CC	Yenesew et al. 1988
Chrysophanic acid (**7**)	*Kniphofia. pumila*	Ethiopia	Rhizomes	SE, TLC and CC	Berhanu et al. 1986
	*Kniphofia reynolds*	Ethiopia	Roots	SE, TLC and CC	Yenesew et al. 1988
	*Kniphofia schimperi*	Ethiopia	Flowers, rhizomes	SE, TLC and CC	Berhanu et al. 1986
Islandicin (**8**)	*Kniphofia foliosa*	Ethiopia	Roots, leaves, and flowers	SE, TLC and CC	Berhanu et al. 1986; Yenesew et al. 1988
	*Kniphofia insignis*	Ethiopia	Rhizomes	SE, TLC and CC	Berhanu et al. 1986
	*Kniphofia isoetifolia*	Ethiopia	Rhizomes	SE, TLC and CC	Berhanu et al. 1986
	*Kniphofia pumila*	Ethiopia	Rhizomes	SE, TLC and CC	Berhanu et al. 1986
	*Kniphofia schimperi*	Ethiopia	Rhizomes	SE, TLC and CC	Berhanu et al. 1986
	*Kniphofia linearifolia*	Ethiopia	Roots	SE, TLC and CC	Yenesew et al. 1988
Islandicin (**8**)	*Kniphofia reynolds*	Ethiopia	Roots	SE, TLC and CC	Yenesew et al. 1988
	*Kniphofia thomsonii*	Kenya	Roots	SE, TLC and CC	Achieng 2009
Helminthosporin (**9**)	*Kniphofia insignis*	Ethiopia	Roots	BGE, TLC and CC	Tadesse et al. 2021
**Dimeric anthraquinones**					
Asphodelin (**10**)	*Kniphofia albescens*	South Africa	Roots	SE, TLC and CC	Van Wyk et al. 1995
	*Kniphofia insignis*	Ethiopia	Roots	BGE, TLC and CC	Tadesse et al. 2021
	*Kniphofia isoetifolia*	Ethiopia	Roots	SE, TLC and CC	Meshesha et al. 2017
	*Kniphofia ensifolia*	South Africa	Whole parts	BGE, SE, TLC and HPLC	Dai et al. 2014
Microcarpin (**11**)	*Kniphofia ensifolia*	South Africa	Whole parts	BGE, SE, TLC, and HPLC	Dai et al. 2014
	*Kniphofia reflexa*	Ethiopia	Rhizomes	FC, CC, HPLC and PTLC	Sema et al. 2018
Chrysalodin (**12**)	*Kniphofia foliosa*	Ethiopia	Leaves	SE, TLC and CC	Dagne et al. 1987
Kniphofine (**13**)	*Kniphofia foliosa*	Ethiopia	Rhizomes	SE, TLC and CC	Berhanu et al. 1985
	*Kniphofia insignis*	Ethiopia	Rhizomes	SE, TLC and CC	Berhanu et al. 1985
	*Kniphofia isoetifolia*	Ethiopia	Rhizomes	SE, TLC and CC	Berhanu et al. 1985
	*Kniphofia pumila*	Ethiopia	Rhizomes	SE, TLC and CC	Berhanu et al. 1985
	*Kniphofia schimperi*	Ethiopia	Rhizomes	SE, TLC and CC	Berhanu et al. 1985
	*Kniphofia ensifolia*	South Africa	Whole parts	SE, TLC and CC	Dai et al. 2014
10-Hydroxy-10-(chrysophanol-7′-yl)-chrysophanol anthrone (**14**)	*Kniphofia foliosa*	Ethiopia	Roots	SE, TLC and CC	Abdissa et al. 2013
	*Kniphofia thomsonii*	Kenya	Roots	SE, TLC and CC	Achieng 2009
10-Hydroxy-10-(chrysophanol-7′-yl)-chrysophanol anthrone (**14**)	*Kniphofia insignis*	Ethiopia	Roots	BGE, TLC and CC	Tadesse et al. 2021
	*Kniphofia isoetifolia*		Roots	SE, TLC and CC	Meshesha et al. 2017
10-Hydroxy-10-(chrysophanol-7′-yl)-chrysophanol anthrone (**14**)	*Kniphofia ensifolia*	South Africa	Whole parts	BGE, SE, TLC, and HPLC	Dai et al. 2014
Chryslandicin (**15**)	*Kniphofia caulescens*	Ethiopia	Roots		Yenesew et al. 1988
	*Kniphofia foliosa*	Ethiopia	Roots	SE, CC, TLC and HPLC	Yenesew et al. 1988; Wube et al. 2005
Chryslandicin (**15**)	*Kniphofia linearifolia*	Ethiopia	Roots	SE, TLC and CC	Yenesew et al. 1988
	*Kniphofia ensifolia*	South Africa	Whole parts	BGE, SE, TLC, and HPLC	Dai et al. 2014
Kniphofione A (**16**)	*Kniphofia ensifolia*	Ethiopia	Whole parts	BGE, SE, TLC, and HPLC	Tadesse et al. 2021
Kniphofione B (**17**)	*Kniphofia ensifolia*	Ethiopia	Whole parts	BGE, SE, TLC, and HPLC	Dagne et al. 1987
10, 10′-Bichrysophanolanthrone (**18**)	*Kniphofia thomsonii*	Kenya	Roots	SE, TLC and CC	Achieng 2009
10-Hydroxy-10-(chrysophanol-7′-yl)-aloe-emodin anthrone (**19**)	*Kniphofia thomsonii*	Kenya	Roots	SE, TLC and CC	Achieng 2009
10-Hydroxy-10-(islandicin-7′-yl)-aloe-emodin anthrone (**20**)	*Kniphofia thomsonii*	Kenya	Roots	SE, TLC and CC	Achieng 2009
10-Methoxy-10, 7′-(chrysophanol anthrone)-chrysophanol (**21**)	*Kniphofia foliosa*	Ethiopia	Roots	SE, TLC and CC	Abdissa et al. 2013
10-(Chrysophanol-7′-yl)-10-(ξ)-hydroxychrysophanol-9-anthrone (**22**)					
**Phenyl anthraquinones and Anthrones**					
Isoknipholone (**23**)	*Kniphofia foliosa*	Ethiopia	Stems	SE, TLC and CC	Yenesew et al. 1994
Isoknipholone anthrone (**24**)	*Kniphofia foliosa*	Ethiopia	Stems		Yenesew et al. 1994
Knipholone anthrone (**25**)	*Kniphofia foliosa*	Ethiopia	Stems	SE, TLC and CC	Dagne and Yenesew 1993; Yenesew et al. 1994
Knipholone cyclooxanthrone (**26**)	*Kniphofia foliosa*	Ethiopia	Roots	SE, TLC and CC	Abdissa et al. 2013
Knipholone (**27**)	*Kniphofia albescens*	Ethiopia	Roots	SE, TLC and CC	Dagne and Yenesew 1993
	*Kniphofia brachystachya*	Ethiopia	Roots	SE, TLC and CC	Dagne and Yenesew 1993
Knipholone (**27**)	*Kniphofia foliosa*	Ethiopia	Roots, leaves, stems, flowers, rhizomes and fruits	SE, TLC, CC and PTLC	Dagne and Steglich 1984; Yenesew et al. 1988; Dagne and Yenesew 1993; Yenesew et al. 1994; Alebachew et al. 2021
	*Kniphofia insignis*	Ethiopia	Rhizomes	SE, TLC and CC	Berhanu et al. 1986
	*Kniphofia isoetifolia*	Ethiopia	Rhizomes	SE, TLC and CC	Berhanu et al. 1986
	*Kniphofia pumila*	Ethiopia	Rhizomes, flowers, roots	SE, TLC and CC	Berhanu et al. 1986; Abdissa et al. 2020
	*Kniphofia schimperi*	Ethiopia	Rhizomes	SE, TLC and CC	Berhanu et al. 1986
	*Kniphofia acraea*	Ethiopia	Roots	SE, TLC and CC	Yenesew et al. 1988
Knipholone (**27**)	*Kniphofia caulescens*	Ethiopia	Roots	SE, TLC and CC	Yenesew et al. 1988
	*Kniphofia flammula*	Ethiopia	Roots	SE, TLC and CC	Yenesew et al. 1988
	*Kniphofia linearifoila*	Ethiopia	Roots	SE, TLC and CC	Yenesew et al. 1988
	*Kniphofia thomsonii*	Kenya	Roots	SE, TLC and CC	Achieng 2009
Knipholone (**27**)	*Kniphofia reflexa*	Ethiopia	Rhizomes	FC, CC, HPLC and PTLC	Sema et al. 2018
**Oxanthrones**					
Foliosone (**28**)	*Kniphofia foliosa*	Ethiopia	Stem	SE, TLC and CC	Yenesew et al. 1994
Isofoliosone (**29**)	*Kniphofia foliosa*	Ethiopia	Stem	SE, TLC and CC	Yenesew et al. 1994
**Organic acids**					
Citric acid (**30**)	*Kniphofia burchelli*	South Africa	Leaves	SE, TLC and CC	Van Oudtshoorn 1964
Malic acid (**31**)	*Kniphofia burchelli*	South Africa	Leaves	SE, TLC and CC	Van Oudtshoorn 1964
Quinic acid (**32**)	*Kniphofia uvaria*	Tokyo, Japan	Leaves	SE, TLC and CC	Yoshida et al. 1975
Shikimic acid (**33**)	*Kniphofia uvaria*	Tokyo, Japan	Leaves	SE, TLC and CC	Yoshida et al. 1975
**Naphthalene Derivatives**					
2- Acetyl-1 -hydroxy-8-methoxy-3- methylnaphthalene (**34**)	*Kniphofia foliosa*	Ethiopia	Roots	SE, CC and HPLC	Wube et al. 2005
2- Acetyl-1 -hydroxy-8-methoxy-3- methylnaphthalene (**34**)	*Kniphofia reflexa*	Ethiopia	Rhizomes	FC, CC, HPLC and PTLC	Sema et al. 2018
Hydroxydeoserone (3,5,8-tri-hydroxy-2-methylnaphthalen-1,4-dione) (**35**)	*Kniphofia isoetifolia*	Ethiopia	Roots	SE, TLC and CC	Meshesha et al. 2017
Dianellin (**36**)	*Kniphofia foliosa*	Ethiopia	Roots, rhizomes	SE, TLC, CC and PTLC	Abdissa et al. 2013; Alebachew et al. 2021
Kniphofiarexine (**37**)	*Kniphofia reflexa*	Ethiopia	Rhizomes	FC, CC, HPLC and PTLC	Sema et al. 2018
2-Acetyl-1,8-dimethoxy-3-methylnaphthalene (**38**)	*Kniphofia reflexa*	Ethiopia	Rhizomes	FC, CC, HPLC and PTLC	Sema et al. 2018
**Indane Derivative**					
Kniphofiarindane (**39**)	*Kniphofia reflexa*	Ethiopia	Rhizomes	FC, CC, HPLC and PTLC	Sema et al. 2018
**Miscellaneous compounds**					
Flavoglucin (**40**)	*Kniphofia thomsonii*	Kenya	Roots	SE, TLC and CC	Achieng 2009
3′′′,4′′′- Dehydroflavoglaucin (**41**)	*Kniphofia thomsonii*	Kenya	Roots	SE, TLC and CC	Achieng 2009
4,6-Dihydroxy-2 methoxyacetophenone (**42**)	*Kniphofia foliosa*	Ethiopia	Stems	SE, TLC and CC	Yenesew et al. 1994
2′,4′,6′-Trimethoxyacetophenone (**43**)	*Kniphofia reflexa*	Ethiopia	Rhizomes	FC, CC, HPLC and PTLC	Sema et al. 2018
3,4-Dihydroxybenzoic acid (**44**)	*Kniphofia reflexa*	Ethiopia	Rhizomes	FC, CC, HPLC and PTLC	Sema et al. 2018

BGE = bioassay guided extraction, CC = column chromatography, FC = flash chromatography, SE = successive extraction, HPLC = high column chromatography, PTLC = preparative thin layer chromatography, TLC = thin layer chromatograph

#### Pre-anthraquinones

Pre-anthraquinones are precursors of anthraquinones, and when treated with a base, they readily convert to the equivalent anthraquinones (Yenesew et al. 1994). From the stem of *K. foliosa*, only two related pre-anthraquinones, aloesaponol III (**27**) and aloesaponol III-8-methyl ether (**28**), have been reported (Yenesew et al. 1994).

#### Monomeric anthraquinones

*Kniphofia* is known for producing monomeric anthraquinones. Monomeric anthraquinones were isolated in many parts of the *Kniphofia* species, including rhizomes, leaves, flowers, roots, and fruits. Seven monomeric anthraquinones (**3–9**) have been found in 11 different *Kniphofia* species so far. Only two monomeric anthraquinones were studied for their pharmacological properties. Helmantosporine (**9**) was isolated from the acetone fraction of *Kniphofia insignis* roots and tested for antibacterial and antifungal properties (Tadesse et al. 2021). The anti-inflammatory potential of the compound chrsophanol (**5**), which was obtained from the methanol crude extract fraction of *Kniphofia reflexa* rhizome, was also investigated (Sema et al. 2018).

#### Dimeric anthraquinones

*Kniphofia* has been shown to be an excellent source of dimeric anthraquinones. In this genus, phenol-oxidative coupling dimerisation of two identical anthraquinones as well as mixed dimerisation has been observed. Dimeric anthraquinones were discovered in several parts of the *Kniphofia* species, including rhizomes, leaves, roots, and whole plants. To date, 12 dimeric anthraquinones (**10–22**) have been found in 11 different *Kniphofia* species. Only 5 dimeric anthraquinones were studied for their pharmacological properties. The antibacterial and antifungal properties of asphodeline (**10**) derived from the acetone fraction of *Kniphofia insignis* roots were investigated (Tadesse et al. 2021). Microcarpin (**11**) was isolated from the rhizomes of a methanol extract of *Kniphofia reflexa* and tested for cytotoxicity on the LLC-MK2 Monkey Kidney Epithelial cell line using the MTT assay with Gleevec (Imatinib) as a positive control. It was found to be moderately cytotoxic with a CC_50_ value of 11.24 μg/mL (Sema et al. 2018). The antimalarial and antiproliferative properties of chyslandicin (**15**), which was isolated from whole parts of an ethanolic crude extract of *Kniphofia ensifolia*, were investigated (Dai et al. 2014). The antiplasmodial activity of the compound 10-methoxy-10, 7′-(chrysophanol anthrone)-chrysophanol (**21**) isolated from the methanol crude extract of *Kniphofia foliosa* roots was investigated, and it showed good activity with IC_50_ values of 1.17 and 4.07 μg/mL, respectively, against chloroquine resistant (W2) and chloroquine sensitive (D6) *P. falciparum* strains (Abdissa et al. 2013). The compound, 10-(chrysophanol-7′-yl)-10-(ξ)-hydroxychrysophanol-9-anthrone (**22**) isolated from dichloromethane extract of *Kniphofia foliosa* roots was evaluated *in vitro* against the chloroquine-sensitive 3D7 strain of *P. falciparum*, and it significantly inhibited malaria parasite development with an ED_50_ value of 0.26 μg/mL (Sema et al. 2018).

#### Phenyl anthraquinones and anthrones

The phenylanthraquinones and anthrones, which are made up of a 1, 8-dihydroxyanthraquinone and an acetylphloroglucinol component linked by a biaryl axis, are another interesting and emerging class of secondary metabolites generated by the *Kniphofia* genus. Compounds **23**, **24**, and **25** were isolated from *Kniphofia foliosa* stem parts, whereas compound **26** was isolated from *Kniphofia foliosa* root parts. Knipholone (**27**) was found in practically all *Kniphofia* species in all parts of the plant, including leaves, rhizomes, stems, roots, and flowers. Only two compounds (**25** and **27**) were tested for their pharmacological properties. Knipholone anthrone (**25**) was tested for its antimalarial, antioxidant, and anti-HIV-1 properties (Habtemariam 2007; Feilcke et al. 2019; Richard et al. 2020). Knipholone (**27**) has a variety of pharmacological properties, including antibacterial, antimalarial, anti-inflammatory, anti-HIV-1, anti-leukotriene, and cytotoxic properties (Wube et al. 2006; Habtemariam 2010; Sema et al. 2018; Feilcke et al. 2019; Abdissa et al. 2020; Alebachew et al. 2021).

#### Oxanthrones

Two oxanthrone compounds (**28** and **29**) were isolated in the stem of *Kniphofia foliosa* by Yenesew et al. (1994). The pharmacological effects of both of these compounds have not been investigated.

#### Organic acids

Another essential component of the *Kniphofia* genus is organic acids. Compounds (**30** and **31**) were isolated in the leaves of *Kniphofia burchelli* and (**32** and **33**) in the leaves of *Kniphofia uvarica*, respectively (Van Oudtshoorn 1964; Yoshida et al. 1975). The pharmacological actions of the compounds have not been studied.

#### Naphthalene derivatives

Naphthalene, commonly known as naphthene, naphthalin, camphor tar, and white tar, is an organic compound having the formula C_10_H_8_. A fused pair of benzene rings makes up the structure of naphthalene. Naphthalene shows various antagonistic activities including anticancer, antimicrobial, anti-inflammatory, antiviral, antihypertensive, antidiabetic, anti-neurodegenerative, antipsychotic, anticonvulsant and antidepressant (Makar et al. 2019). *Kniphofia* species roots and rhizomes were used to isolate naphthalene derivatives. Three different *Kniphofia* species have been reported to contain five naphthalene derivatives (**34–38**). The pharmacological activities of three of these compounds (**34**, **36**, and **37**) were investigated. On the LLC-MK2 Monkey Kidney Epithelial Cell Line, the cytotoxic activity of the compound isolated from methanolic crude extract of *Kniphofia reflexa* rhizomes was tested against Gleevec (Imatinib) as the positive control using the MTT assay. The compound (**34**) was extremely cytotoxic, with a CC_50_ of 4.43 μg/mL (Sema et al. 2018). The antimalarial activity of a compound (**36**) isolated from 80% methanol rhizome extracts of *Kniphofia foliosa* was evaluated in mice against the chloroquine (CQ) sensitive ANKA strain of *Plasmodium berghei*. At a dose of 200 mg/kg body weight, the compound dianellin (**36**) exhibited a substantial suppression value of 60.16%, and it extended the treatment group's mean survival time (Alebachew et al. 2021). Kniphofiarexine (**37**) was isolated from crude methanol extracts of *Kniphofia reflexa* rhizome and evaluated for anti-inflammatory effects on phagocyte oxidative burst. After activation, phagocytic cells released free reactive oxygen species (ROS) radicals, which were quantified using a luminal-enhanced chemiluminescence assay with ibuprofen as a positive control. Kniphofiarexine (**37**) was less effective than the reference drug ibuprofen in inhibiting monocyte activity, with just 42.2% compared to 73.2% for ibuprofen (Sema et al. 2018).

#### Indane derivative

Kniphofiarindane (**39**) is the only indane derivative that has been isolated from *Knifolia* species so far. On the LLC-MK2 Monkey Kidney Epithelial Cell Line, the cytotoxic activity of the compound isolated from methanol crude extract of *Kniphofia reflexa* rhizomes was evaluated against Gleevec (Imatinib) as the positive control using the MTT assay. The compound (**39**) was moderately cytotoxic, with a CC_50_ of 16.35 μg/mL (Sema et al. 2018).

#### Miscellaneous compounds

So far, four miscellaneous compounds (**40–44**) have been isolated from three *Kniphofia* species: *Kniphofia thomsonii*, *Kniphofia foliosa*, and *Kniphofia reflexa*. These compounds' biological activities have not been studied.

### Pharmacological activities

**Figure 3.**
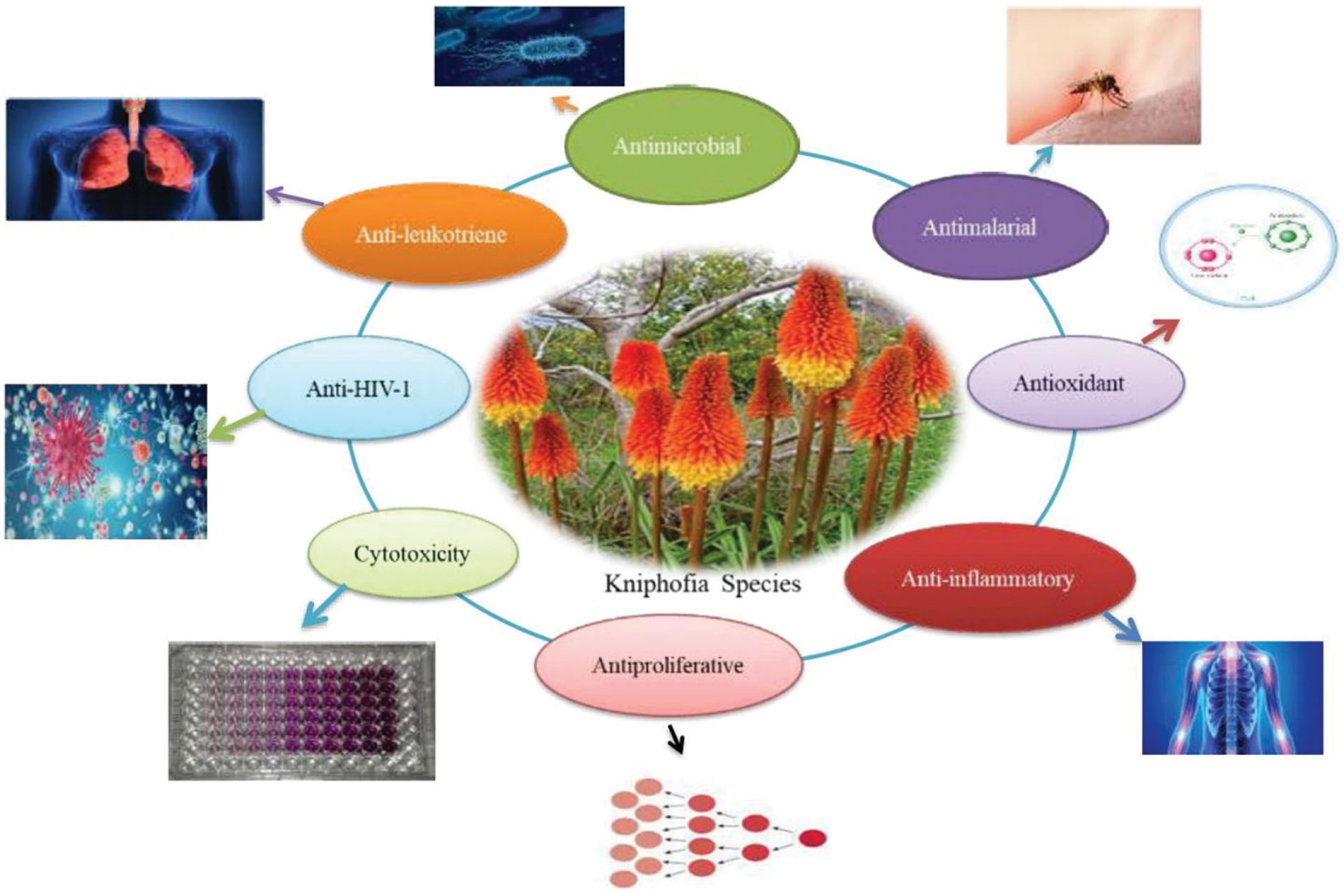
Graphical representation of pharmacological activity reports in *Kniphofia* species.

Modern and traditional approaches to healthcare frequently coexist and complement one another. Ethnomedicinal practices are now widely used in the search for novel pharmaceuticals (Gurib-Fakim 2006). Recent interest in examining plant constituents for their pharmacological activity and screening for useful and safe phytochemicals has renewed (Nigussie et al. 2021). Dysmenorrhoea, eczema, malaria, gonorrhoea, Hepatitis B, Blood purifier, gout, and cervical and breast cancer are just a few of the ailments for which *Kniphofia* species have been used in traditional medicine (Table 1). Various *in vitro* and *in vivo* pharmacological activities of *Kniphofia* species such as antibacterial, antifungal, antimalarial, antioxidant, anti-inflammatory, anti-HIV-1, anti-leukotriene, antiproliferative and cytotoxic activity are showed in Figure 3 and mentioned below.

#### Antibacterial activity

The acetone crude extracts of *Kniphofia pumila* roots were tested for antibacterial activity against *E. coli*, *K. pneumonia*, and *S. aureus* using the agar disc diffusion method with gentamycin as a positive control. The extract demonstrated inhibition zones of 12.6, 11.8 and 10.7 mm, which are very similar to the positive control gentamycin, which has inhibition zones of 16, 18 and 13 mm, respectively (Abdissa et al. 2020). The reported compound knipholone (**27**) also exhibited against *E. coli*, *S. auerus* and *S. typhimurium* with zones of inhibition 14, 16 and 12 mm, respectively. Whereas, the combined crude extracts with ZnCl_2_, as well as the reported compound with ZnCl_2_, had much higher antibacterial activity against *E. coli* and *S. aureus* bacterial strains than the crude extract and isolated compound, which could be attributed to a synergetic effect (Abdissa et al. 2020). The antibacterial activity of acetone crude extracts of *Kniphofia insignis* roots was examined using the agar disc diffusion method with gentamycin as a positive control against *E. coli*, *P. aeruginosa*, *S. aureus*, and *B. subtilis*. The inhibition zones of the crude extract were 18, 14, 15, and 18 mm, which are fewer activities than the inhibition zones of the positive control gentamycin, which were 32, 22, 33, and 31 mm, correspondingly. The reported compounds helmantosporine (**9**) and asphodeline (**10**) were found to inhibit Gram-positive and Gram-negative bacteria with zones of inhibition ranging from 11 to 15 mm, with helmantosporine (**9**) having the maximum activity (15 mm) against *P. aeruginosa* (Tadesse et al. 2021). Using the agar disc diffusion method and gentamicin as a positive control, Meshesha et al. (2017) investigated the antibacterial activity of methanol-chloroform (1:1 v/v) and ethyl acetate extracts of *Kniphofia isoetifolia* roots against *S. aureus*, *E. faecalis*, *P. aeruginosa*, and *E. coli*. The results showed that the crude extracts had significant activity on both Gram-positive and Gram-negative bacterial strains, with zones of inhibition ranging from 21 to 28 mm and potencies that were closely related between the two crude extracts. However, the ethyl acetate extract exhibited highest zone of growth inhibition (28 mm) on *E. coli* and *E. faecalis* (Meshesha et al. 2017). The reported compounds asphodeline **(10)** and 10-hydroxy-10, 7′-(chrysophanolanthrone) chrysophanol **(14)** had strong inhibitory activities against the examined bacterial strains (inhibition zone diameters ranging from 18 to 30 mm), with asphodeline, 10-hydroxy-10,7′-(chrysophanolanthrone) chrysophanol having the best inhibitory capacity 30 and 28 mm respectively, that is extra comparable with standard drug having 31 mm inhibition zone (Meshesha et al. 2017).

#### Antifungal activity

Using the agar disc diffusion method and chlotrimazole as a positive control, the antifungal activity of acetone crude extracts of *Kniphofia insignis* roots was investigated against *Fusarium spp*. The inhibition zones of the crude extract were 18 mm, which are quite similar to the inhibition zones of the positive control chlotrimazole, which were 20 mm. Helmantosporine (**9**) and asphodeline (**10**) were also shown to be effective against *Fusarium spp*., with zones of inhibition of 12 and 13 mm, respectively (Tadesse et al. 2021).

#### Antimalarial activity

Using malaria SYBR Green I-based *in vitro* assay techniques with reference drugs chloroquine and mefloquine, study examined antiplasmodial activity of methanol root extracts of *Kniphofia foliosa* against chloroquine resistant (W2) and chloroquine sensitive (D6) strains of *P. falciparum*. The crude extract showed IC_50_ values of 11.28 and 8.92 μg/mL, which were weaker than the reference drugs chloroquine (0.22 and 0.01 μg/mL) and mefloquine (0.03 and 0.003 μg/mL respectively). However, compound 10-methoxy-10,7′-(chrysophanol anthrone)-chrysophanol (**21**) demonstrated good activity, with IC_50_ values of 1.17 and 4.07μg/mL, respectively (Abdissa et al. 2013). *Kniphofia foliosa* 80% methanol rhizome extracts were tested in mice for antimalarial activity against the chloroquine (CQ) sensitive ANKA strain of *Plasmodium berghei*. At dosages of 400 and 200 mg/kg body weight, respectively, the highest activities were suppressed with 61.52 and 51.39% suppression. Furthermore, when compared to the negative controls, the extract considerably increased the survival days of the treated groups at those levels. The reported compound, knipholone (**27**) and dianellin (**36**) were likewise shown to have significant suppression values of 55.14 and 60.16% at doses of 100 and 200 mg/kg, respectively, and they extended the treatment groups' mean survival days (Alebachew et al. 2021).

Using chloroquine as a positive control, the antiplasmodial activity of dichloromethane extracts of *Kniphofia foliosa* roots were tested *in vitro* against the chloroquine-sensitive 3D7 strain of *P. falciparum*. The crude extract showed antiplasmodial action with an ED_50_ of 3.8 μg/μmL, which is a low level of activity when compared to the reference drug, which had an ED_50_ of 0.0075 μg/mL (Wube et al. 2005). Also, the compound 10-(chrysophanol-7′-yl)-10-(ξ)-hydroxychrysopanol-9-anthrone (**22**) which was isolated from the roots and tested for antimalarial activity *in vitro*, inhibited the malaria parasite *P. falciparum*'s development significantly, with ED_50_ values of 0.260 μg/mL (Wube et al. 2005). The antiplasmodial activity of the compound chryslandicin (**15**), reported from the whole parts of ethanol extract of *Kniphofia ensifolia*, was tested using the SYBR Green I assay with artemisinin as the reference drug against the Dd2 chloroquine-resistant strain of *P. falciparum*. With IC_50_ values of 0.2 μM, the compounds displayed comparable antiplasmodial action to the reference drug artemisinin, which has an IC_50_ value of 0.007 μM (Dai et al. 2014). Similarly, Feilcke et al. (2019) studied that knipholone (**27**) and knipholone anthrone (**25**) isolated from *Kniphofia foliosa* have substantial antimalarial activity, with IC_50_ values of 1.9 and 0.7 μM, respectively, against *P. falciparum* 3D7 strain using chloroquine as a reference drug, which has an IC_50_ value of 0.005 μM.

#### Antioxidant activity

An *in vitro* assay was used to test the antioxidant activity of the knipholone anthrone (**25**) reported from *Kniphofia foliosa* against 2, 2-diphenyl-1-picrylhydrazyl (DPPH) radicals, the compound has a concentration-dependent scavenging effect. In the DPPH assay, the compound and the positive control (-)-epicatechin (EC) had IC_50_ values of 22 and 8.7 μM, respectively. It was also more effective than EC at scavenging superoxide anions and inhibiting hydroxyl radical degradation of deoxyribose. The compound appeared to form a complex with Fe^2+^, had a concentration-dependent reducing power, and protected isolated DNA from damage induced by Fenton reaction-generated hydroxyl radicals (at concentrations of 4.4 μM and higher) (Habtemariam 2007).

#### Anti-inflammatory activity

The anti-inflammatory effects of crude methanol extracts of *Kniphofia reflexa* rhizome and its reported compounds kniphofiarexine (**37**), knipholone (**27**), and chrysophanol (**5**) on phagocyte oxidative burst were investigated. Phagocytic cells released free reactive oxygen species (ROS) radicals (oxidative burst) after activation, which was measured using a luminal-enhanced chemiluminescence assay with ibuprofen as a positive control. Compounds knipholone (**27**), and chrysophanol (**5**) (concentration 100.10 μg/mL) reduced the zymosan-induced oxidative burst in polymorpho-neutrophils (PMNs) moderately, with CC_50_ values of 38.7 μg/mL and 20.0 μg/mL, respectively, as compared to the positive control, which had a CC_50_ value of 27.16 μg/mL. The crude extract and compound kniphofiarexine **(37)** were less effective in inhibiting monocyte activity, with just 42.4% and 42.2%, respectively, compared to 73.2% for the reference drug ibuprofen (Sema et al. 2018).

#### Antiproliferative activity

The antiproliferative activity of the compound chryslandicin (**15**), which were isolated from the whole parts of the ethanol extract of *Kniphofia ensifolia*, was tested using the Alamar blue assay. When compared to the positive control actinomycin/taxol, the compound demonstrated modest antiproliferative action with IC_50_ values of 4 μM (Dai et al. 2014).

#### Cytotoxic activity

Habtemariam (2010) evaluated the cytotoxicity activity of knipholone (**27**) and knipholone anthrone (**25**) reported from *Kniphofia foliosa* in leukemic and melanocyte cancer cell lines using the annexin V-FITC apoptosis assay. Both compounds had anticancer action, with knipholone anthrone generating a quick onset of cytotoxicity with IC_50_ values ranging from 0.5 to 3.3 μg/mL. When comparing the cytotoxicity of both compounds, knipholone was 70–480 times less harmful to cancer cells. The cytotoxicity of knipholone anthrone was also linked to a rapid loss of membrane integrity, resulting in necrotic cell death, according to morphological and biochemical analyses (Habtemariam 2010). The cytotoxicity of 80% methanol root extracts of *Kniphofia foliosa* against ten human cancer cell lines MCF-7, A427, RT-4, SiSo, LCLC-103H, DAN-G, A2780, KYSE-70, HL-60, and U-937 was evaluated using crystal violet cell proliferation and MTT cell viability assays. *Kniphofia foliosa* root extracts reduced cell growth in all cell lines tested, with IC_50_ values ranging from 14.54 to 27.06 μg/mL (Tesfaye et al. 2021). The acute toxicity of *Kniphofia foliosa* methanol rhizome extracts and its reported compounds knipholone (**27**) and dianellin (**36**) was investigated. The LD_50_ of extracts and reported compounds were found to be greater than 2000 mg/kg in the study (Alebachew et al. 2021). The MTT technique was used to test the cytotoxic activity of the methanolic crude extract of *Kniphofia reflexa* rhizomes and their chemical constituents against Gleevec (Imatinib) as the positive control on the LLC-MK2 Monkey Kidney Epithelial Cell Line. With a CC_50_ of 4.43 μg/mL, 2-acetyl-1-hydroxy-8-methoxy-3-methylnaphthalene (**34**) was highly cytotoxic. Compounds kniphofiarindane (**39**) and microcarpin (**11**) were moderately cytotoxic as well, with CC_50_ values of 16.35 μg/mL and 11.24 μg/mL, respectively. The crude extract as well as the other compounds kniphofiarexine, knipholone and chrysophanol (**37, 27** and **5**) were non-cytotoxic (Sema et al. 2018). Using the Alamar blue assay, the dichloromethane extract of *Kniphofia foliosa* roots was tested for cytotoxic action on KB cells against podophyllotoxin as the reference drug. The crude extract had an ED_50_ value of 35.2 μg/mL, which indicates low cytotoxic activity when compared to the reference drug, which had an ED_50_ value of 0.0123 μg/mL (Wube et al. 2005). In addition, the compound 10-(chrysophanol-7′-yl)-10-(ξ)-hydroxychrysopanol-9-anthrone (**22**) which was isolated from the roots and tested for cytotoxicity, has a very low toxicity with an ED_50_ of 104 μg/mL (Wube et al. 2005).

#### Anti-HIV-1 activity

The anti-HIV-1 capability of knipholone (**27**) and knipholone anthrone (**25**) derived from *Kniphofia foliosa* was tested in HIV-1c infected peripheral blood mononuclear cells. At concentrations of 0.5, 5, 15, and 50 μg/mL, knipholone anthrone demonstrated considerable growth inhibition (HIV-1c replication suppression) of more than 60%, according to the study (Feilcke et al. 2019). Also, study showed that knipholone anthrone (**25**), induces both HIV-RNA and HIV-protein in primary cells from HIV infected donors (Richard et al. 2020).

#### Anti-Leukotriene activity

Activated human neutrophil granulocytes, as well as 12-LO, COX-1, and COX-2 tests, were used to study the leukotriene inhibitory action of knipholone (**27**) obtained from the root dichloromethane extract of *Kniphofia foliosa*. With an IC_50_ value of 4.2 μM, knipholone demonstrated the ability to be a selective inhibitor of leukotriene biosynthesis when compared to the positive control, zileuton, which had an IC_50_ value of 10.4 μM (Wube et al. 2006) (Table 3). 

**Table 3.** Summary of Pharmacological studies of isolated compounds from *Kniphofia* species.

Activity	Plant species	Plant part	Extract	Isolated compound	Method (mode of action)	Effect	Ref
Antibacterial	*Kniphofia pumila*	Root	Acetone	Knipholone (**27**)	Agar disc diffusion/*in vitro*	The compound knipholone showed inhibition zones of 14, 16 and 12 mm against *E. coli*, *S. auerus*, and *S. typhimurium*, which are very similar to the positive control gentamycin has inhibition zones of 16, 18 and 13 mm, respectively.	Abdissa et al. 2020
Antibacterial	*Kniphofia insignis*	Root	Acetone	Helmantosporine (**9**) and Asphodeline (**10**)	Agar disc diffusion/*in vitro*	The compounds helmantosporine and asphodeline were found to have zones of inhibition ranging from 11 to 15 mm against *E. coli*, *P. aeruginosa*, *S. aureus*, and *B. subtilis*, which are lower activities than the inhibition zones of the positive control gentamycin, which were 32, 22, 33, and 31 mm, respectively.	Tadesse et al. 2021
Antibacterial	*Kniphofia isoetifolia*	Root	Ethyl acetate	Asphodeline (**10**) 10-hydroxy-10,7′-(chrysophanolanthrone) chrysophanol (**14**)	Agar disc diffusion/*in vitro*	Asphodeline and 10-hydroxy-10, 7′-(chrysophanolanthrone) chrysophanol are effective against *S. aureus*, *E. coli*, *E. faecalis* and *P. aeruginosa*, with inhibition zones ranging from 18 to 30 mm, which is comparable to gentamicin has 31 mm inhibition zone.	Meshesha et al. 2017
Antifungal	*Kniphofia insignis*	Root	Acetone	Helmantosporine (**9**) and Asphodeline (**10**)	Agar disc diffusion/*in vitro*	Helmantosporine and asphodeline have inhibition zones of 12 and 13 mm, respectively, against *Fusarium spp.*, which are less effective than the positive control chlotrimazole has an inhibition zone of 20 mm.	Tadesse et al. 2021
Antimalarial	*Kniphofia* *foliosa*	Root	Methanol	10-methoxy-10,7′-(chrysophanol anthrone)-chrysophanol (**21**)	SYBR Green I/*in vitro*	The compounds demonstrated good activity against chloroquine resistant (W2) and chloroquine sensitive (D6) strains of *P. falciparum*, with IC_50_ values of 1.17 and 4.07μg/mL, respectively	Abdissa et al. 2013
Antimalarial	*Kniphofia foliosa*	Rhizome	Methanol	Knipholone (**27**) and Dianellin (**36**)	blood-induced CQ resistant rodent parasite in mice*/in vivo*	The compound had significant suppression values of 55.14 and 60.16 % against the chloroquine (CQ) sensitive ANKA strain of *Plasmodium berghei* at dosages of 100 and 200 mg/kg, respectively.	Alebachew et al. 2021
Antimalarial	*Kniphofia foliosa*	Root	Dichloromethane	10-(chrysophanol-7′-yl)-10-(ξ) hydroxychrysopanol-9-anthrone (**22**)	SYBR Green I/*in vitro*	The compound 10-(chrysophanol-7′-yl)-10-(ξ)-hydroxychrysopanol-9-anthrone inhibited chloroquine-sensitive 3D7 strain of *P. falciparum* development significantly, with ED_50_ values of 0.260 μg/mL	Wube et al. 2005
Antimalarial	*Kniphofia ensifolia*	Whole parts	Ethanol	Chryslandicin (**15**)	SYBR Green I/*in vitro*	The compound had IC_50_ values of 0.2 μM against Dd2 chloroquine-resistant *P. falciparum*, which was comparable to the reference drug artemisinin, which has an IC_50_ value of 0.007 μM.	Dai et al. 2014
Antimalarial	*Kniphofia foliosa*	Root	Ethyl acetate	Knipholone (**27**) and Knipholone anthrone (**25**)	*P. falciparum* maintained in continuous culture in human erythrocytes/*in vitro*	The compound IC_50_ values of 1.9 and 0.7 μM, respectively, against *P. falciparum* 3D7 strain using chloroquine as a reference drug, which has an IC_50_ value of 0.005 μM.	Feilcke et al. 2019
Antioxidant	*Kniphofia foliosa*	Root	Methanol	Knipholone anthrone (**25**)	DPPH radical assay/*in vitro*	The compound and the positive control (-)-epicatechin (EC) had IC_50_ values of 22 and 8.7 μM, respectively	Habtemariam 2007
Antioxidant	*Kniphofia foliosa*	Root	Methanol	Knipholone anthrone (**25**)	DPPH radical assay/*in vitro*	The compound and the positive control (-)-epicatechin (EC) had IC_50_ values of 22 and 8.7 μM, respectively	Habtemariam 2007
Anti-inflammatory	*Kniphofia reflexa*	Rhizome	Methanol	Knipholone **(27),** and Chrysophanol **(5)**	Luminal-enhanced chemiluminescence assay/*in vitro*	Compounds knipholone **(27),** and chrysophanol **(5)** (concentration 100.10 μg/mL) reduced the zymosan-induced oxidative burst in polymorpho-neutrophils (PMNs) moderately, with CC_50_ values of 38.7 μg/mL and 20.0 μg/mL, respectively, as compared to the positive control ibuprofen, which had a CC_50_ value of 27.16 μg/mL	Sema et al. 2018
Antiproliferative	*Kniphofia ensifolia*	Whole part	Ethanol	Chryslandicin (**15**)	Alamar blue assay/*in vitro*	The compound demonstrated modest antiproliferative action with IC_50_ values of 4 μM	Dai et al. 2014
Anti-Leukotriene	*Kniphofia foliosa*	Root	Dichloromethane	Knipholone (**27**)	COX-1, and COX-2 tests/*in vitro*	With an IC_50_ value of 4.2 μM, knipholone demonstrated the ability to be a selective inhibitor of leukotriene biosynthesis when compared to the positive control, zileuton, which had an IC_50_ value of 10.4 μM	Wube et al. 2006
Anti-HIV-1	*Kniphofia foliosa*	Root	Ethyl acetate	Knipholone anthrone (**25**)	HIV-1c infected peripheral blood mononuclear cells/*in vitro*	At concentrations of 0.5, 5, 15, and 50 μg/mL, knipholone anthrone demonstrated considerable growth inhibition of more than 60 %	Feilcke et al. 2019
Cytotoxic	*Kniphofia foliosa*	Root	Methanol	Knipholone anthrone (**25**) and knipholone (**27**)	Annexin V-FITC apoptosis assay/*in vitro*	Both compounds had anticancer action, with knipholone anthrone generating a quick onset of cytotoxicity with IC_50_ values ranging from 0.5 to 3.3 μg/mL	Habtemariam 2010
Cytotoxic	*Kniphofia foliosa*	Rhizome	Methanol	Knipholone (**27**) and dianellin (**36**)	Wister rats/*in vivo*	The LD_50_ of compounds were found to be greater than 2000 mg/kg	Alebachew et al. 2021
Cytotoxic	Kniphofia reflexa	Rhizome	Methanol	Microcarpin (**11**), Kniphofiarexine (**37**), Knipholone (**27**), Chrysophanol (**5**), 2-acetyl-1-hydroxy-8-methoxy-3-methylnaphthalene (**34**) and kniphofiarindane (**39**)	MTT assay/*in vitro*	With a CC_50_ of 4.43 μg/mL, 2-acetyl-1-hydroxy-8-methoxy-3-methylnaphthalene (**34**) was highly cytotoxic. Compounds kniphofiarindane **(39)** and microcarpin **(11)** were moderately cytotoxic as well, with CC_50_ values of 16.35 μg/mL and 11.24 μg/mL, respectively. other compounds kniphofiarexine, knipholone and chrysophanol (**37, 27** and **5**) were non-cytotoxic	Sema et al. 2018
Cytotoxic	*Kniphofia foliosa*	Root	Dichloromethane	10-(chrysophanol-7′-yl)-10-(ξ)-hydroxychrysopanol-9-anthrone (**22**)	Alamar blue assay/*in vitro*	The compound showed very low toxicity with an ED_50_ of 104 μg/mL as compared to the reference drug podophyllotoxin, which had an ED_50_ value of 0.0123 μg/mL	Wube et al. 2005

## Future perspective

Natural-source drugs are gaining popularity because they are less expensive, have fewer or no side effects, and are better tolerated by patients. Plants provide an alternate source of active secondary metabolites for drug development (Beshah et al. 2020). *Kniphofia* species have multiple ranges of ethnomedicine uses in the treatment of different diseases. The pharmacological activities and toxicological consequences of the extracts from these plants have also been reported. Few members of this genus, however, have been studied. Secondary metabolites generated from plants have limited biological activity. As a result, a revival of interest in the *Kniphofia* species phytochemistry and pharmacology could lead to the development of lead drugs. In this regard, a random clinical trial as well as the pharmacokinetics of these plants could provide the possibility of developing effective curative agents. This requires isolating bioactive metabolites and pharmacological activities from plant extracts, as well as conducting clinical trials, pharmacokinetics, and toxicological analyses.

## Conclusions

In this review, we outline what we know about botany, ethnomedicinal uses, reproduction, conservation, phytochemistry, and pharmacological activity of *Kniphofia* species. *Kniphofia* species were traditionally used to treat gonorrhoea, malaria, hepatitis B, blood purifier, wounds, cervical and breast cancer, and many other ailments, according to the findings. In addition, the *Kniphofia* species has been utilised as an ornamental plant, pollen and nectar sources for honeybees, and a pollution indicator. It is used in horticulture and is grown in both home and botanical gardens. It has been noticed that all studied plants belong to the same genus, they have a number of common pharmacological actions, such as antibacterial, antimalarial, and cytotoxic activity. The major compounds isolated from the majority of *Kniphofia* species are monomeric anthraquinone, dimeric anthraquinone, and phenyl anthraquinones and anthrones. The genus afforded exemplary drug leads such as knipholone (**27**) and knipholone anthrone (**25**) with anti-HIV-1, anti-leukotriene, anti-inflammatory, antimalarial and cytotoxicity activity. Nevertheless, given the presence of the genus in the red data list of South Africa and its broad range of pharmacological activities, greater attention should be dedicated to it. Further investigation should be conducted to evaluate promising cruds extracts as well as compounds in search for new drug candidates.
